# Biodegradable Materials for Bone Repair and Tissue Engineering Applications

**DOI:** 10.3390/ma8095273

**Published:** 2015-08-31

**Authors:** Zeeshan Sheikh, Shariq Najeeb, Zohaib Khurshid, Vivek Verma, Haroon Rashid, Michael Glogauer

**Affiliations:** 1 Faculty of Dentistry, Matrix Dynamics Group, University of Toronto, 150 College Street, Toronto, ON M5S 3E2, Canada; 2 School of Clinical Dentistry, University of Sheffield, Sheffield S10 2TN, UK; E-Mail: shariqnajeeb@gmail.com; 3 School of Materials and Metallurgy, University of Birmingham, Birmingham B15 2TT, UK; E-Mail: drzohaibkhurshid@gmail.com; 4 Biomaterials Department of Biomedical Engineering, School of Engineering, King Faisal University, Al-Hofuf 31982, Saudi Arabia; 5 Faculty of Dentistry, Division of Biomedical Sciences, McGill University, 2001 McGill College Avenue, Montreal, QC H3A 1G1, Canada; E-Mail: vivek.verma@mail.mcgill.ca; 6 College of Dentistry, Division of Prosthodontics, Ziauddin University, 4/B, Clifton, Karachi 7550, Pakistan; E-Mail: drh.rashid@hotmail.com; 7 Matrix Dynamics Group, Faculty of Dentistry, University of Toronto, 150 College Street, Toronto, ON M5S 3E2, Canada; E-Mail: Michael.Glogauer@utoronto.ca

**Keywords:** biomaterials, biodegradable materials, bone regeneration, bone repair, tissue engineering

## Abstract

This review discusses and summarizes the recent developments and advances in the use of biodegradable materials for bone repair purposes. The choice between using degradable and non-degradable devices for orthopedic and maxillofacial applications must be carefully weighed. Traditional biodegradable devices for osteosynthesis have been successful in low or mild load bearing applications. However, continuing research and recent developments in the field of material science has resulted in development of biomaterials with improved strength and mechanical properties. For this purpose, biodegradable materials, including polymers, ceramics and magnesium alloys have attracted much attention for osteologic repair and applications. The next generation of biodegradable materials would benefit from recent knowledge gained regarding cell material interactions, with better control of interfacing between the material and the surrounding bone tissue. The next generations of biodegradable materials for bone repair and regeneration applications require better control of interfacing between the material and the surrounding bone tissue. Also, the mechanical properties and degradation/resorption profiles of these materials require further improvement to broaden their use and achieve better clinical results.

## 1. Introduction

Bone is a composite natural living tissue which comprises of an organic phase in which calcium containing inorganic phase crystals are embedded [1]. Bone by weight contains about 30% matrix, 60% mineral and 10% water [2]. The bone matrix is primarily collagen which responsible for the tensile strength. The mineral component of bone is calcium phosphate, which imparts compressive strength to the bone tissue [3]. There are two types of bone tissue, cortical (compact), and cancellous (trabecular). Compact bone has Young’s modulus of elasticity ranging from 17–20 GPa and compressive strength in the range of 131–224 MPa [2,4], while Young’s modulus and compressive strength for trabecular bones are 50–100 MPa and 5–10 MPa respectively [2,4].

Bone tissue is susceptible to fracture as a result of trauma, pathology and resorption [5,6]. Bone fixation and repair devices traditionally are fabricated with metals and used clinically [7,8]. Stainless steel, titanium and its alloys have been employed for the majority of fracture fixation treatments [9,10]. However these metallic devices and implants are not biodegradable and often require a second surgery in order to remove these from the body [10,11,12]. This not only increases the hospitalization time and health care cost but also elevates chances of infection and complications. Also, due to the mismatch between the mechanical properties of these devices and the natural bone, mechanical forces and loads are retained by implants and are not transferred to the healing bone [13]. This is termed as “stress shielding” which results in unwanted bone resorption and implant loosening [13,14,15,16]. Bone defect management involves using autologous bone graft which is harvested from various sites of the patient body [17,18]. Autologous bone grafting is considered as the gold standard and it possesses all the characteristics necessary for new bone growth, *i.e.*, (i) osteoconductivity (scaffold to promote bone apposition) [19]; (ii) osteogenicity (containing osteoprogenitor cells) [20] and (iii) osteoinductivity (provide signals to induce osteogenic differentiations of local stem cells) [21,22]. However, there are limitations and concerns of this approach such as, limited bone supply, donor site morbidity, anatomical, structural and surgical limitations and increased bone resorption during healing [23,24,25,26,27]. Other biological sources, such as allograft and xenogenic bone has also been evaluated and used with varying clinical success for bone repair and regeneration [6,28]. The use of synthetic materials (alloplasts) is another way to repair and regenerate lost bone tissue [29,30].

According to the degradation performance, materials for bone repair can be classified into two groups: bio-inert and biodegradable materials [31,32]. The bio-inert materials have been used widely for clinical use with success; they do have some problems. For example, they are mostly inert implants that stay in human body forever until removed surgically. A major drive for continued research to develop biodegradable materials is the need for new materials with properties tailored to meet the biochemical and biomechanical requirements of bone tissue engineering [31,32,33,34]. The basic concept is that the substitute biomaterial acts as a scaffold for the surrounding cells/tissue to invade, grow, and thus guide tissue regeneration towards new bone formation [35,36,37,38,39]. Once bone repair and healing has occurred, scaffold removal via *in vivo* degradation is desirable both from a clinical and a biomechanical point of view. Therefore, biodegradable materials are sought since they can be used as an implant and do not require a second surgical event for removal [40,41]. The biodegradable materials must support the bone tissue regeneration and repair process while providing mechanical support and degrading to non-toxic products ultimately being removed by the body [42]. While providing a brief introduction to chemistry and properties of major classes of materials, the main aim of this review is to provide the readers with an update on recent developments in different classes of biodegradable materials for bone repair applications. 

## 2. Biodegradable Materials

There are a variety of biomaterials that have been researched upon and used clinically for bone repair and regeneration applications [43]. The degradation of implant materials is accompanied with an unwanted decrease in mechanical properties. However, if the degradation is controlled and gradual, then the loads will transfer from the implants to bone tissue and soft tissues to avoid the stress shield effect [15,42]. The development of biodegradable rods, plates, pins, screws and suture anchors has progressed in recent years. Biodegradable polymers, ceramics and metals are the main three kinds of widely studied and clinically used biodegradable materials (Table 1). In this section, these biodegradable materials are reviewed and recent advancements summarized.

**Table 1:** Physical properties of natural bone tissue compared with other degradable and non-degradable materials and their applications [2,4,9,44,45,46,47,48,49,50].

Material Type	Compressive Strength (MPa)	Tensile Strength (MPa)	Young’s Modulus (GPA)	Elongation (%)	Degradation Time (Months)	Loss of Total Strength (Months)	Applications for Bone Repair and Regeneration
***A. Bone***							
* Human cortical	131–224	35–283	17–20	1.07–2.10	NBR	none	Autograft and allograft used for defect filling, alveolar ridge augmentation, sinus
* Human cancellous	5–10	1.5–38	0.05–0.1	0.5–3	NBR	0.5–1	augmentation, dental ridge preservation [51,52,53,54,55,56,57,58,59,60]
***B. Degradable***							
* Collagen	0.5–1	50–150	0.002–5	3	2–4	1–4	Carriers (sponges) for BMP [61,62,63], composite with HA [64], membranes for GBR [65,66], scaffolds [67]
* Chitosan	1.7–3.4	35–75	2–18	1–2	4–6	<3	Scaffolds, microgranules, composite materials, VBA, membranes, xerogels [68,69,70,71,72]
* PGA	340–920	55–80	5–7	15–20	3–4	1	Internal fixation, graft material, scaffold, composite [73,74,75]
* PLLA	80–500	45–70	2.7	5–10	>24	3	Carrier for BMP, scaffolds, composite with HA [76,77,78,79,80,81,82]
* D,L(PLA)	15–25	90–103	1.9	3–10	12–16	4	Fracture fixation, interference screws [83,84,85]
* L(PLA)	20–30	100–150	2.7	5–10	>24	3	Fracture fixation, Interference screws, scaffolds, bone graft material [74,77,86,87,88,89]
* PLGA	40–55	55–80	1.4–2.8	3–10	1–12	1	Interference screws, microspheres and carriers for BMP, scaffolds, composite [90,91,92,93]
* PCL	20–40	10–35	0.4–0.6	300–500	>24	>6	Scaffolds and composites with HA fillers [94,95,96,97,98,99]
* Hydroxyapatite	500–1000	40–200	80–110	0.5–1	>24	>12	Scaffolds, composites, bone fillers (granules and blocks), pastes, vertebroplasty, drug delivery, coatings [100,101,102,103,104,105,106,107,108,109,110,111]
* TCP	154	25–80	60–75	1–2	>24	1–6	Bone fillers, injectable pastes, cements [112,113,114,115,116,117,118,119,120,121,122]
* Brushite	35–60	15–25	40–55	2–3	>24	1–6	Drug delivery, restoration of metaphyseal defects, ligament anchor, reinforcement of
* Monetite	15–25	10–15	22–35	3–4	3–6	1–3	Osteosynthesis screws, ridge preservation, vertical bone augmentation, defect filling, vertebroplasty [123,124,125,126,127,128,129,130,131,132,133,134,135,136,137,138,139,140,141,142,143]
* Magnesium	65–1000	135–285	41–45	2–10	0.25	<1	Implants, osteosynthesis devices, plates, screws, ligatures, and wires [122,144,145,146,147,148,149,150,151,152,153,154,155,156,157,158]
***C. Non-Degradable***							
* Titanium alloy	900	900–1000	110–127	10–15	No	None	Implants, plates, screws, BMP carriers, orthognathic surgery, mid-facial fracture treatment [159,160,161,162,163,164,165,166]
* Stainless Steel	500–1000	460–1700	180–205	10–40	No	None	Implants, plates, mini–plates, screws [167,168,169,170]
* Bioglass	40–60	120–250	35	0–1	No	None	Bone defect fillers [171,172,173,174,175,176,177]

NBR: Natural bone remodeling; PGA: Poly glycolic acid; PLLA: Poly L-lactic acid; PLGA: Poly lactic glycolic acid; PCL: Poly caprolactone; PLA: Poly lactic acid; PEO: Poly ethylene oxide; BMP: bone morphogenetic proteins; GBR: guided bone regeneration; VBA: vertical bone augmentation; HA: hydroxyapatite.

### 2.1. Polymers

Polymers are macromolecules that are composed of covalently bonded repeating monomers that can be same or different, *i.e.*, homopolymers and copolymers [178]. These materials can be amorphous and crystalline with chains being linear, branched or cross-linked with other chains [179]. Polymer properties are affected by temperature and it is important to synthesize biodegradable polymers with the glass transition temperature (*T*_g_) above the body temperature as polymers become very flexible above their defined *T*_g_ [178].

Biodegradable polymers are one of the primary and common biomaterials used for bone repair and tissue engineering. Their biodegradability and controlled degradation rates are highly beneficial for clinical applications [180,181]. The degradation of polymeric materials can be altered by changing their structural composition and fabrication techniques [179]. The degradation process and rate is affected by various factors such as the molecular composition molecular weight (Mw) and crystallinity [80,182]. The types of monomers making up the polymeric material affect the sensitivity of hydrolysable bonds [9]. The longer the polymer chains are the more hydrolytic chain scissions are required to obtain biodegradation. Since crystallinity is the measure of organization, interactions and packing in a material affects biodegradation, more crystalline materials possess stronger inter- and intra-molecular bonding therefor degrade slowly when compared to amorphous polymers [42].

An optimal interaction on a cellular and biochemical level is required for a positive outcome to be achieved towards the formation of a functional tissue [79]. There are a few criteria for biodegradable polymers in order to be used successfully for bone repair and tissue engineering applications: (i) the polymer surface should allow for cell adhesion and growth to occur; (ii) post implantation *in vivo*, there should be no inflammatory or toxic response towards the polymer or its degradation products; (iii) have sufficiently high porosity that is interconnected; (iv) have high surface area and adequate space for extracellular matrix; (v) be completely degradable with controlled resorption timing of the scaffold matrix (degradation rate ideally matching with the regenerating bone tissue); and lastly (vi) the polymeric material should allow reproducible processing into three dimensional (3D) structures [35,79,183].

Based on their origin, polymers can be classified as natural or synthetic. Due to their inherent low strength, natural polymers are mainly used for the repair of small bone fractures that do not impart high loads onto the implant materials. As for the synthetic polymers, by controlling the design and synthesis, polymers with improved mechanical properties can be prepared [184,185]. Synthetic polymers also have the advantage of having a well-controlled and reproducible molecular structure and are also non-immunogenic.

#### 2.1.1. Natural Biodegradable Polymers

##### Collagen

Collagen is the most abundant protein present in the human body and is the major component in bone and skin tissues [186,187]. Collagen is a polymer with repeating sequences having a molecular weight (*M*_w_) of 300,000 and a chain length of 300 nm. The repeating sequences of collagen are responsible for the helical structure and inherent mechanical strength [188]. Due to the fact that collagen undergoes enzymatic degradation in the body, the mechanical and biological properties of collagen have been thoroughly studied for biomedical applications. The collagen rate of degradation can be controlled and altered introducing cross-linking in the polymer chains and also by enzymatic pre-treatments [189].

Collagen when used as a biomaterial is biocompatible, biodegradable and osteoconductive [190,191]. Collagen can be processed into different forms such as tubes, sheets, nano-fiber matrices, foams, powders and viscous solutions and dispersions that are injectable [79]. Human bone is a mineral/organic natural composite consisting of hydroxyapatite (HA) and collagen (mainly). Hence, composites produced using calcium phosphates and collagens are considered as the most biomimetic system for osseous replacement and regenerative applications [192,193,194,195,196]. Calcium phosphate particles when mixed with collagen result in easily moldable biomaterials for clinical use [197]. Collagen coatings on calcium phosphate substrates and implants have been shown to facilitate and enhance early cell adhesion and proliferation [198,199]. This results in increased osteoconduction, osteointegration and bone formative capacity of these materials when implanted *in vivo* [198,199]. For these particular advantages, calcium phosphate and collagen containing composite materials have been developed [200] via particle-gel mixing and powder compression methods [201,202,203].

Additives such as citric acid when are added to collagen and used to set dicalcium phosphate dehydrate (DCPD) also known as brushite, it is observed that the speed setting reaction is increased significantly and the hardened biomaterials has compressive strength similar to cancellous bone (48.0 MPa) [44]. Addition of citric acid also increases the workability of the collagen-brushite cement paste and enables the mixing of high collagen gel (3 wt %) with the cement powders. With this combination using citric acid, the setting time was shortened along with a decrease in viscosity which enhances injectibility of the composite [204]. The effect of introducing cross-linking into the collagen-brushite composite phase was also investigated by immersion of the biomaterial into gluteraldehyde solution (2%). However, this immersion was not shown to increase the compressive strength of the composite materials [44].

##### Chitosan

Chitosan is a natural biopolymer derived from chitin. It is a linear polysaccharide, composed of glucosamine and *N*-acetyl glucosamine in a particular ratio [205]. The molecular weight of chitosan may range from 300 to 1000 kDa depending on its source and processing methods. Although chitosan is generally insoluble in aqueous solutions above pH 7, but when placed in diluted acids having pH less than 6, the protonated free amino group of glucosamine facilitates the solubility of the material [206,207,208]. Chitosanase, papain and lyzozyme are known to degrade chitosan *in vitro* [161]. The *in vivo* degradation takes place primarily due to lyzozyme and is regulated via hydrolysis of the acetylated residues. The chitosan degradation rate depends on the level of crystallinity and acetylation of the polymer [209]. The chemical alteration of chitosan polymer can affect degradation and solubility rate significantly and the highly deacetylated form demonstrates slow biodegradation occurring over several months *in vivo* [209].

Chitosan is biocompatible and can be molded to form structures and scaffolds having porous micro-architecture which promotes osteoconduction [210]. Chitosan with calcium phosphate have been researched upon for this purpose. Scaffolds containing high molecular weight chitosan demonstrated superior mechanical properties compared with scaffolds constructed with medium molecular weight chitosan [210]. Chitosan/nano-crystalline calcium phosphate based scaffolds have rough surface and ~20 times greater surface area per unit mass than chitosan scaffolds alone [211]. This increase in roughness and surface area results in greater protein adsorption, cell attachment and proliferation for bone regeneration and repair applications [211]. Also, these scaffolds have better mechanical properties which can be attributed to the better dispersion and strong interaction of calcium phosphate nano-crystals with chitsoan [211]. Collagen has been incorporated into chitosan to form composite micro-granules to be used as bone substitutes. The micro-granular structure allows for close packing into defects and the interconnected pores in between the micro-granules allow for new bone and vascular ingrowth [68,69]. The addition of filler particles such as HA to these chitosan/collagen composite biomaterials improves mechanical strength significantly [70].

Chitosan membranes fabricated with silica xerogels have been evaluated for applications in guided bone regeneration (GBR) with regards to bone regeneration ability [212]. Significantly enhanced new bone formation has been observed using the chitosan/slica xerogel membranes compared with pure chitosan membranes alone [71]. Also, after 3 weeks the histomorphometric analysis revealed that the defect was completely healed with the hybrid membrane, whereas on ~57% of defect closure was observed with chitosan membrane used alone [212]. In another study sulfated chitosan with varied sulfate groups, sulfur content and molecular weight were investigated to see the effect of being tailored on bioactivity of bone morphogenetic protein 2 (BMP-2) [213]. Osteoblast differentiation was stimulated *in vitro* and ectopic bone formation was induced *in vivo* by low dose of synthetic sulfated chitosan [213].

#### 2.1.2. Synthetic Biodegradable Polymers

The most extensively researched upon synthetic biodegradable polymers are Poly (α-hydroxy acids) also known as polyesters. These synthetic polymers can be synthesized from a wide range of monomeric units via ring opening and condensation polymerization methods. Poly (hydroxyl acid) has an ester bond that is cleaved by hydrolysis which results in a reduction in the molecular weight (*M*_w_) of the polymer [214]. However, this reduction in *M*_w_ does not decrease the mass of the implant materials. The rate of degradation of polyesters is dependent on the exposed surface area, crystallinity, initial *M*_w_ and the ratio between hydroxyl ions and the monomers (in copolymers) [186].

The most extensively investigated and used polymers among the poly (α-hydroxy acid) class are the poly (glycolic acid) (PGA), poly (lactic acid) (PLA) and their copolymer poly (lactic-co-glycolide) (PLGA) [43,215,216,217,218]. Apart from PGA, these polymers are soluble a variety of organic solvents and hence can be processed by many solvent and thermal-based methods [219,220]. These polymers are considered to be suitable candidates for bone repair and regeneration applications since they are biocompatible with and biodegradable in the human body [187]. The biodegradation is mediated via hydrolytic degradation through the process of de-esterification and the removal of monomeric byproducts takes place through natural excretory pathways [220,221]. Through the method of esterification all polyesters, theoretically, can be made degradable. However, it is a chemically reversible process and only aliphatic chains between ester bonds can degrade in the time that is required in order to be useful for biomedical applications [214].

Implantable devices for internal fixation for fracture repair have been fabricated using these polymers and have gained popularity [222]. They first generated interest three decades ago when polyesters were utilized for suture materials and still remain one of the widely used synthetic biodegradable polymers [223]. When polyesters are used alone for the fabrication of devices the mechanical properties of highly porous scaffolds are relatively weak than that required for bone tissue engineering applications [224]. They also lower the local pH *in vivo* due to the degradation products that in turn accelerates the degradation rate of the implants to an extent that limits their clinical usefulness [225]. Another disadvantage of this rapid disintegration is that the acidic degradation byproducts (monomeric or oligomeric hydroxyl-carboxylic acids) induce an inflammatory reaction [226,227,228].

##### Poly (Glycolic Acid)

**Figure 1:**
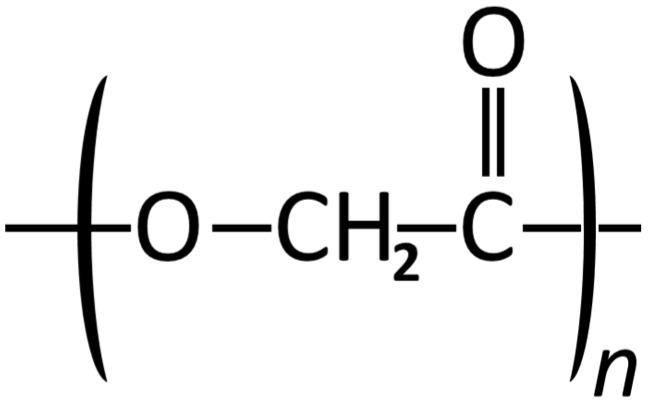
Structural formula of Poly glycolic acid.

Poly (glycolic acid) (PGA) is a highly crystalline synthetic polymer (45%–50% crystallinity) of glycolic acid (Figure 1). Due to the high crystallinity, melting point (>200 °C), tensile modulus and controlled solubility, PGA was first employed for clinical use as sutures and as biomedical implants [229]. PGA has a high degradation rate due to its hydrophilic nature and the mechanical strength of PGA after implantation for 14 days usually decreases by 50% and by ~90% after 28 days [47]. The degradation product of PGA is hydroxyacetic acid and is either metabolized by the liver (as CO_2_ and H_2_O as final products) or discharged through the kidneys via the urine [230]. Biodegradation, no aggregation and lack of cytotoxic response are the main advantages of using PGA as a degradable biomaterial [231,232].

PGA has been used as a self-reinforced foam and is stiffer (Young’s modulus of 12.5 GPa) [233] than other degradable polymers for clinical use [73]. Also, PGA loses its mass in 6–12 months due to *in vivo* degradation [79]. PGA has been evaluated as a biomaterial for fabrication of devices used for internal fixation of bone [74,234]. Since PGA loses its strength after implantation with time, this limits their usefulness for load bearing fractured segments [79]. PGA has also been reinforced with amorphous carbonated-apatite and used as a bone replacement graft material but it was observed that this material was only useful in small defects or non-loading bearing situations [75].

##### Poly (Lactic Acid)

**Figure 2:**
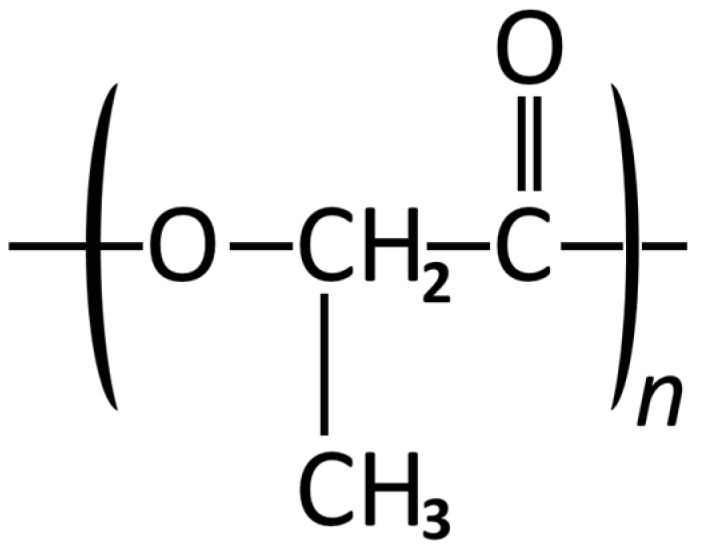
Structural formula of Poly lactic acid.

Poly (lactic acid) (PLA) was first used for medical applications as sutures and rods for the treatment of mandibular fractures in dogs [235], and since has been researched upon extensively [236,237,238]. PLA is aliphatic thermoplastic polyester with linear polymeric chains and undergoes *in vivo* biodegradability via enzymatic and hydrolytic pathways [239,240,241,242] (Figure 2). PLA has excellent mechanical and thermal properties, is biocompatible and biodegradable [243] and has a renewable source [239] which makes it affordable and available for biomedical applications. Lactic acid is a chiral molecule and exists as two stereoisometric forms which result in distinct polymers based on morphology such as l-PLA, d-PLA, d,l-PLA and meso-PLA [79]. l-PLA and d-PLA are stereoregular, d,l-PLA is a racemic polymer (mixture of l- and d-lactic acid), and meso-PLA is obtained from d,l-lactide. Crystalline l-PLA that is resistant to hydrolysis [244,245] and amorphous d,l-PLA that is more sensitive to hydrolysis [233] are mostly used for clinical applications [246,247,248]. *In vivo*, the Lactic acid that is released by PLLA degradation is converted into glycogen in the liver or incorporated into the tricarboxylic acid cycle and excreted from the lungs as water and carbon dioxide [9].

Scaffolds fabricated for bone tissue engineering applications require specific material properties (porous architecture, adequate porosity levels and mechanical strength) and therefore L-PLA in preferred in the orthopedic applications because it satisfies most of these requirements [74,77,86,87]. Poly (l-lactic acid) (PLLA) has been investigated as a biomaterial and fabricated into scaffolds [88,249,250] by utilizing salt leaching [251], phase separation [252,253], and gas-induced foaming [77,254] methods. These technologies and methods can be used to fabricate porous polymers having porosity below 200 µm [79]. However, they do not allow control over porosity in the 200–500 µm size range which is imperative for new bone formation and vascular in growth [79]. Precise extrusion manufacturing (PEM) is another method that has been used to produce PLLA scaffold [77]. The scaffold porosity was ~60% but the effectiveness of adequate porosity distribution resulted in improved mechanical properties (~8 MPa compressive strength) [77].

Thermally induced phase separation (TIPS) is another technique that can and has been used successfully to fabricate highly porous scaffolds for bone tissue engineering [79]. This technique utilizes dioxane as a solvent and can be used to create a composite structure having interconnected pores of PLLA with hydroxyapatite (HA). The porosity obtained by this technique can be as high as 95% with pore sizes ranging from few microns to several hundred microns with also an improvement in the mechanical properties (from ~6 MPa for PLLA alone to 11 MPa for the composite) [79]. This composite skeleton of PLLA/HA when implanted was proven to have good bonding to bone structure [77,78]. The compressive strength, porosity levels and distribution and interfacial properties have been improved upon further by using micro and nano sized HA which encourages molecular interactions and formation of chemical linkages between the PLLLA matrix and the inorganic fillers [78,81,255,256,257,258]. Cell culture experiments with mesenchymal stem cells (MSCs) revealed that cell affinity and proliferation was improved greatly with the use of these scaffolds [77,79].

PLA synthetic polymers have also been utilized to create a partially degradable bone graft for supporting weak bone in proximal femur [82]. This biomaterials comprises of an outer elastic layer of d,l-PLA, HA and calcium carbonate with an inner layer of titanium dip-coated into solutions of PLLA with suspended calcium salts. d,l-PLA owing to its fast degradation rate is strategically placed on the outside to promote biodegradation and replacement with new bone tissue. The PLLA degrades slowly and provides the biocompatible interface between the biological tissues and the inert metallic core of the implant which provides the required mechanical stability [79].

##### Poly (Lactide-co-glycolide)

Poly lactide-co-glycolide (PLGA) is formed by the combination of lactic and glycolic acid. l- and d,l- both have been utilized for copolymerization and when used in the compositional range of 25%–75%, forms amorphous PLGA polymer [79]. 50–50% PLGA has been shown to be hydrolytically unstable [179,259]. PLGA used in clinical applications has been shown to be biocompatible, non-cytotoxic and non-inflammatory [260,261]. Although PLGA has been extensive used in a variety of clinical applications, its use is limited in the field of orthopedics [262]. The reason for this is probably the hydrophobic nature of PLGA which does not support cell adhesion for promoting bone in-growth [263]. By altering the unit ratio of lactide to glycolide and the molecular weight (*M*_w_), its biodegradation and mechanical properties can somewhat be controlled [264]. Even with optimization, PLGA is not an ideal candidate to be used for load bearing applications due to the low mechanical strength [79].

PLGA pellets with a lactide to glycolide ratio of 85:15 have been investigated in stimulated body fluid (SBF) to mimic the process of mineralization in teeth and bone *in vitro* [265]. The pore size of this polymer was in the range of 250–450 µm and after 16 days the mineral grown on the surface was a carbonated apatite [265]. Since, this mineral is very close to natural bone tissue it indicates the potential of these biomaterials for bone regeneration applications. Moldable, biodegradable bone graft substitute with PLGA loaded with osteogenic bone morphogenetic protein-2 (BMP-2) microspheres incorporated with calcium phosphate cement (CPC) for bone applications has been investigated [92]. The lactic to glycolic ratio in this polymer/CPC composite was 50:50 and the mechanical strength was very low [266]. Despite lacking adequate mechanical strength, the composite demonstrated good biodegradation rate [92].

Scaffolds constructed with PLGA reinforced with calcium phosphate such as HA as filler improves the mechanical properties compared to scaffold made with PLGA alone. Also, the presence of HA imparts the scaffold with enhanced ability for osteoblast attachment and improved metabolic activity [267,268,269]. *In vitro* cultures have also shown that the addition of HA to polymer matrix result in increased mineralization [270] as there is more surface are and roughness for cell attachment and more inorganic material to support bone in-growth [271,272]. Some studies show that the ideal particle size range is 50–300 µm which promotes bone growth [273] whereas, other studies suggest that porous interconnection of the scaffold is more important [274]. With the presence of PLGA, the mechanical properties can be controlled and biomaterials can be prevented from getting too brittle [79]. Three dimensional (3-D) HA/PLGA porous scaffolds have been created using solvent casting and particulate leaching techniques for use in bone replacement applications [275]. Surface grafting of HA by PLGA matrix deposition has shown improvement in the interfacial properties between the polymer and the inorganic CPC in comparison with the non-grafted HA/PLGA [275]. Although both grafted and non-grafted biomaterials showed similar potential towards enhancing mineralization, the grafted composite exhibited better bone bonding ability [275].

##### Poly (ε-Caprolactone)

**Figure 3:**
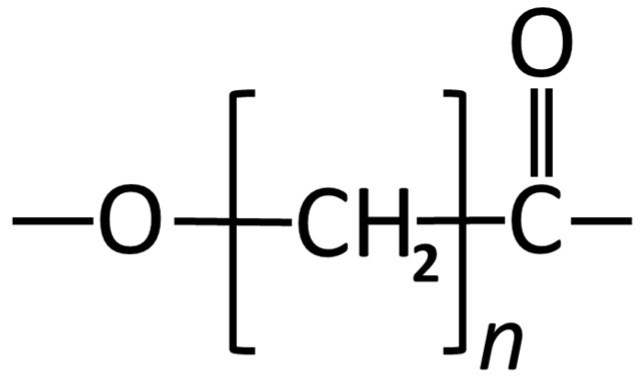
Structural formula of Poly (caprolactone).

Poly(ε-caprolactone) (PCL) is an aliphatic polyester that is a semi-crystalline polyester and can be processed in various forms due to it being highly soluble in a variety of organic solvent [79,276] (Figure 3). PCL is a polymer that has a very high thermal stability when compared with other aliphatic polymers [276,277]. The decomposition temperature (Td) of PCL is 350 °C, while the Td of aliphatic polyesters is usually between 235 °C and 255 °C [278]. 

PCL has been investigated as a biomaterial for orthopedic application [279,280]. PCL is a biodegradable and biocompatible polymer that has been used for bone repair and treatment of bone defects [97,98]. However, PCL has been shown not to be an ideal biomaterial for these purposes due to its slow degradation rate and inferior mechanical properties [281,282]. Melt blending technique has been used to reinforce PCL with HA [94]. By using this method the polymer is fully melted and the HA particles used as reinforcing fillers are dispersed in the polymeric matrix [94,95,96]. The particle size used with this technique to fabricate the composites is very important. It was observed that the HA particles with a size range of 3–8 µm imparted higher compressive strengths to the composite materials [94]. Although the addition of fillers improves the compressive strength, increasing the filler content more than a certain level renders these PCL/HA composites too brittle for clinical use [79].

##### Benzyl Ester of Hyaluronic Acid

Benzyl esters of hyaluronic acid are also known as HYAFF-11 and they demonstrate good rate of degradation and their degradation products are non-toxic [283]. The degradation time varies from 1–2 weeks to 2–3 months and occurs by hydrolysis via ester bonds. The degradation is dependent on the degree of esterification with the de-esterified HYAFF-11 is more soluble and resembles the precursor hyaluronic acid [284,285]. HYAFF-11 has been investigated for use in bone tissue engineering and vascular graft preparation applications [284,286]. HYAFF-11 has been reinforced with α-tricalcium phosphate (α-TCP) to form a hydrogel [286]. The compressive strength was seen to improve from ~3 MPa for pure HYAFF-11 to ~17 MPa for the hydrogel. This increased compressive strength value being closer to cancellous bone strength suggests that these HYAFF-11 based hydrogels can be utilized as bioresorbable bone fillers for orthopedic and oral maxillofacial applications.

##### Poly-*para*-dioxanone

**Figure 4:**
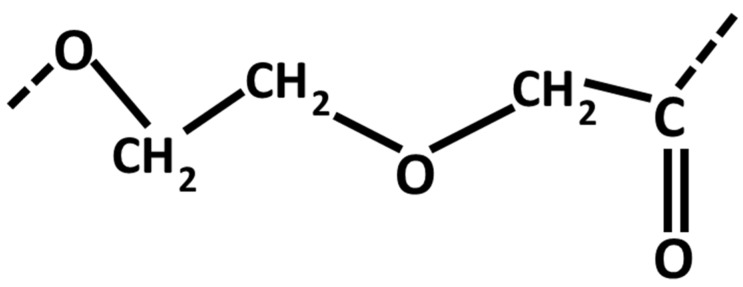
Structural formula of Poly-*para*-dioxanone.

Poly-*para*-dioxanone (PDS) is a polymer consisting of multiple repeating ether-ester units. PDS is obtained by the ring-opening polymerization of *para*-dioxanone monomer [287,288] (Figure 4). PDS is a polyester used in the field of medicine in form of films, laminates, molded products, foams, adhesives and surface coatings [289,290]. Due to its excellent biocompatibility, biodegradation and flexibility, PDS has been investigated for use in tissue regeneration and fracture repair applications [291,292,293]. PDS when used for internal fixation of fractures has been shown to be completely biodegradable within the bone tissues [294,295]. PDS can be resorbed completely *in vivo* within 5–7 months via the alteration of its crystallinity, molecular weight Mw and the melting temperature [47]. 

#### 2.1.3. Polymer Based Composites

Polymeric orthopedic prostheses have been fabricated with pure polymers lack adequate mechanical properties required for stress-bearing long bone fracture stabilization [296]. This encouraged research to be carried out towards the development of polymeric composite materials that would possess satisfactory mechanical and biological properties. Completely resorbable polymer composite materials have been used in oral and maxillofacial surgery [297,298]. However, their poor mechanical properties restricted their use and they could not be used for load-bearing applications. Resorbable polymers (polylactide) and its co-polymers such as PLA, PLGA and PLLA degrade when exposed to body fluid [48,50]. Non-resorbable additives such as polyamide fibers have been used in composites to improve material properties by making them partially resorbable [299,300]. The need for second surgery in to remove these non-resorbable fibers lead to the use of completely resorbable and/or bioceramics as reinforcements in the composite materials. HA/PLA, tricalcium phosphate/PLGA and phosphate glass fiber/PLA are some examples of completely resorbable polymeric composites [301,302,303,304,305]. Fibers, coatings and coupling agents can be added to control the rate of degradation. PGF/PLA composites have been developed and the *in vitro* mechanical and chemical properties have been investigated to develop completely resorbable composites for bone fracture fixation devices [306,307,308,309]. The biodegradation rate of various types of polymeric composites has also been studied [310] and bone plates, screws and intramedullary rods have been developed for application to load-bearing long bone fracture fixation and stabilization [303,305,311,312,313,314,315].

### 2.2. Bioceramics

Ceramic biomaterials were initially investigated and used in the field of orthopedic surgery as an alternative to metallic biomaterials. Bioceramics are currently used for bone defect filling, fracture repair and stabilization and replacement of diseased bone tissues [316,317,318]. Ceramic materials are biocompatible, have corrosion resistance and demonstrate tremendous bioactivity. Disadvantages of bioceramics include poor fracture toughness, brittleness and extremely high stiffness [314]. The strength of degradable bioceramics is significantly lower than that of non-resorbable materials [43,317]. Solution-driven and cell-mediated processes are considered to responsible for degradation of bioresorbable ceramics [319]. Lamellar bone replacement occurs after cellular degradation of the ceramic matrix has taken place. The biological behavior of bioceramics is dependent on the physical characteristics and chemical composition [317,320].

#### 2.2.1. Tricalcium Phosphate

**Figure 5:**
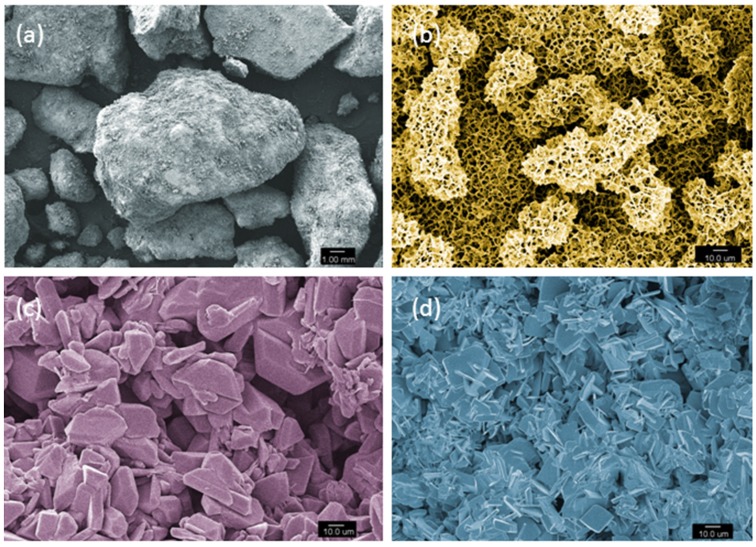
Scanning electron microscope micrographs of (**a**) β-Tricalcium phosphate granules; 50× magnification; (**b**) Hydroxyapatite, 5000× magnification; (**c**) Dicalcium phosphate dihydrate crystals, 5000× magnification; (**d**) Dicalcium phosphate anhydrous crystals, 5000× magnification.

Tricalcium phosphate (TCP) is a resorbable and bioactive ceramic material (Figure 5a). TCP has two crystalline forms: 1. α-TCP and 2. β-TCP and the crystallinity and chemical composition resembles closely to that of the mineral phase of bone tissue [44]. TCP demonstrates a higher rate of biodegradation than hydroxyapatite after implantation *in vivo* [321] which is regulated by a combination of passive dissolution and osteoclast mediated resorption [322]. TCP has been used as synthetic bone defect fillers in dental maxillofacial and orthopedic application [323,324]. TCP demonstrates osteoconductivity and active resorption due to its interconnected microporosity which plays a vital role in the graft-bone complex remodeling process [112,113,114]. Preclinical experiments have shown TCP to almost completely resorb (~95%) after a month and half of implantation in rat tibias with new bone formation and marrow reformation [115]. Similar bone in-growth has been observed for TCP implantation in cancellous bone in canine models [325]. TCP bone replacement grafts have shown to be rapidly infiltrated with bone and slowly resorb by osteoclasts between 6 and 24 months [116].

#### 2.2.2. Hydroxyapatite

Hydroxyapatite (HA) is a bioactive and bioresorbable (variable rate and extent) calcium phosphate that forms the majority of the inorganic component of bone tissue [114,326,327] (Figure 5b). The atomic ratio for calcium to phosphate is 1.67 in HA [44]. Synthetic HA when prepared via a high-temperature reaction is a highly crystalline ceramic. Although synthetic and natural HA differ in terms of physical microstructure, crystal size and porosity, chemical similarities to bone accounts for the osteoconductive potential [114,327]. The bioresorption of HA is slow and heavily related to its properties. Minimal degradation and slow resorption was reported after implantation for 12 weeks in rabbit femoral bone [47]. HA based bioceramics are used for small bone defect filling after tumor resection and/or after bone loss due to fractures in humerus, tibia, calceneus, radius and vertebra [101]. Biphasic ceramic formulation of HA/TCP (60/40) has been shown to provide an intimate scaffold-bone contact, yet has very limited application to be used for load-bearing segmental defects [100].

There have been efforts towards developing HA based bioceramic materials that have been doped with ions. Strontium-HA [102], magnesium-HA [328] and silicon-HA [103] have been tested to improve mechanical and biological properties for bone tissue engineering applications. Although synthetic HA demonstrates good cytocompatibility, its usefulness as a scaffold material is limited due to its moderate to low solubility after implantation [329]. Manganese and zinc doped HA bone substitute materials have been shown to have quicker resorption kinetics [330]. HA has already proven to be an excellent carrier for osteogenic cell populations and osteoconductive growth factors and in future promises to have great utility as a bioactive agent delivery vehicle [104].

#### 2.2.3. Dicalcium Phosphates

Dicalcium phosphates (DCPs) are acidic calcium phosphates having an alkaline calcium source, an acidic phosphate source, water as the main constituents. Sometimes other additives are included in the cement composition to alter the setting time and physical properties. Very basic alkaline sources such as calcium oxide [331] and calcium hydroxide [332] can be used to prepare DCP cements. Dicalcium phosphate dehydrate (DCPD), mineral name brushite (Figure 5c), has a calcium to phosphate (Ca/P) ratio of 1 and hence calcium phosphates with Ca/P ratio higher than 1 can be utilized to make brushites [44,333]. TCP is the most common basic calcium source in brushite cements (Ca/P ratio of 1.5) [334,335]. Phosphoric acid is the simplest source of acidic phosphate ions required to prepare DCP cements [334,336]. Since DCP cements have a Ca/P ratio of 1, so acidic calcium phosphate compounds used to prepare DCP cements need to have a Ca/P ratio lower than 1. The only two calcium phosphates with this low ratio are monocalcium phosphate anhydrous (MCPA), also known as monetite and monoclacium phosphate monohydrate (MCPM) [331,337]. MCPM is more commonly used to prepare DCP cements because it has a water molecule that it donates during the cement setting process [44]. DCPD cements can be used as precursors to the anhydrous form that is DCPA or monetite [44,333] (Figure 5d). Monetite can be obtained by dehydration of preset brushite cements or by altering the setting mechanics to favor DCPA formation [44,338,339].

DCP cement based bioceramics are biodegradable. However, brushite cements after implantation start converting to HA which ultimately limits their total resorption and biodegradation rate [44]. This phase conversion effect has not been observed with monetite biomaterials and they have a greater amount of new bone formation and infiltration associated with them [333]. Resorbable and injectable brushite cements have been investigated for use in treating metaphyseal bone defects [136,340]. Brushite cements have also been used for treatment of fractures in the tibial plateau [136] and distal metaphysic bone [340].

Leakage and dispersion of cement particles into adjacent tissues has been observed and clinically reported, but since the cements are biodegradable they eventually resorb without any serious complications [340]. Stabilization of osteosynthesis screws is vital in achieving successful stabilization in patients suffering from complicated fractures. Traditionally polymethylmethacrylate (PMMA) cements have been used but they have inherent limitations such as being not strong enough, having exothermic setting reaction and its monomer is cytotoxic [341,342,343,344]. For this reason brushite cements have been evaluated and it was found that the pull-out force was increased by 3-fold [131]. Monetite resorbable bioceramics have been evaluated in preclinical and clinical situations for bone augmentation and regeneration in orthopedic and dental applications successfully [125,128,338].

### 2.3. Magnesium Based Biodegradable Materials and Alloys

Elemental magnesium (Mg) was discovered in 1808 and Mg and its alloys have generated significant interest for use in biomedical applications as implants, osteosynthesis devices, ligatures, and wires for aneurysm treatment and connectors for vessel anastomosis [144,345]. Mg^2+^ is a cation that is mostly stored in bone tissues and is the fourth most abundant ion in the human body. Mg based metals corrode in aqueous environments via electrochemical reactions that result in the production of Mg hydroxide and hydrogen gas [144]. The corrosion product of Mg (Mg^2+^) is easily excreted in urine resulting in the good biological behavior observed when Mg and its alloys are used for medical applications [144]. Mg based biomaterials have better mechanical properties when compared with other conventional biodegradable materials such as polymers and ceramics [47]. The density of Mg based metals (1.7–2.0 g/cm^3^) matches closely with the density of bone (1.8–2.1 g/cm^3^) [47]. Whereas, the densities of other metals (titanium and stainless steel) are much higher or much lower as in case of polymers when compared with natural bone tissue [47]. Also, the elastic modulus of Mg based metals is ~45 GPA which is closer to natural bone (Table 1). Titanium alloys and stainless steels used for bone applications have an elastic modulus of ~110 GPa and ~200 GPa respectively [346]. Due to this the stress shielding effect with the use of Mg metallic materials is reduced significantly.

Based on the distinct advantages of Mg based metals, they have been extensively investigated both *in vitro* and *in vivo* for osteologic repair and regeneration applications. Mostly the focus has been on fabricating screws and plates for fracture fixation and porous scaffold [144]. However, since these have inferior mechanical properties than the conventional metallic non-degradable devices, Mg based devices are not being used for load bearing application [47,49,347]. Although these Mg based materials possess a superior strength to weight ratio compared to other biodegradable materials, a critical issue is the controllability of the degradation rates [348]. They have a fast degradation rate which induces osteolysis, hemolysis and rapid reduction of mechanical properties [348,349]. In order to control this fast degradation, many surface modifications have been tried with varying success such as micro-arc oxidation [350,351], anodization [352], phosphating [353,354], electro-deposition [355] and biomimetic treatment [356].

Binary magnesium-calcium (Mg-Ca) alloys with various levels of calcium contents under different processing conditions have been investigated [357]. Owing to the low density of calcium (1.55 g/cm^3^), the Mg-Ca alloys have similar density to bone [358]. The binary Mg-Ca alloys are generally composed of two phases: (i) the α-Mg and (ii) Mg_2_Ca. An increase in the α-Mg phase in the alloy microstructure leads to higher corrosion rates whereas hot extrusion and hot rolling reduces the corrosion [359]. After implantation of Mg-Ca alloy pins in rabbit femoral shafts no cytotoxicity was observed and elevated activity of osteocytes and osteoblasts was shown around the implants indicating good biocompatibility and bioactivity [360].

Zinc (Zn) is an element that provides a strengthening effect (280 MPa tensile strength) [361,362] and improves corrosion resistance when incorporated into Mg alloys [361]. Mg alloys with 6% Zn have been shown to degrade *in vivo* with a degradation rate of 2.32 mm per year and not be cytotoxic to L-929 cells [361]. Other binary Mg alloys with aluminium (Al), Manganese (Mg), Indium (In), Silver (Ag) and Zirconium (Zr) added to their microstructure have been researched upon to evaluate their biological behavior [351]. Further *in vivo* experimentation and long term implantation studies are required to determine the effect of these elemental inclusions on corrosion resistance, biodegradation and mechanical strength before being applied to clinical applications in the future.

## 3. Biocompatibility of Implantable Materials and Their Degradation Products

To perform successfully, implantable biomaterials must not cause abnormal responses in local tissues and should not produce toxic or carcinogenic effects. Biodegradable materials in particular should serve their intended function while releasing products of degradation that are biocompatible and do not interfere with tissue healing [43]. A major concern associated with using biodegradable materials especially polymers is the possibility of local inflammation due to themselves or via their degradation products [363]. Various polymers have been used successfully for clinical use in the form of sutures, and researchers have theorized that these materials can also be used as fixation devices or replacement implants in orthopaedic and maxillofacial applications [364]. Once implanted, the biodegradation and resorption process begins and are accompanied by a release of acidic by-products which can result in inflammatory reactions [365]. If the capacity of the surrounding tissue to eliminate the by-products is low, due to the poor vascularization or low metabolic activity, the chemical composition of the by-products may lead to local and systemic disturbances [366].

PGA polymers are generally considered to be immunologically inert and not much evidence of infection or symptomatic foreign body reaction exists with their uses as self-reinforced rods [87]. However, in cytological analysis of materials aspirated from malleolar fracture repair effusions developed around PGA implants, inflammatory monocytes have been observed [367,368,369]. Also, in a series of clinical study of PGA, used for fracture fixation in the foot, foreign body reactions were often reported [369]. In some cases, osteolytic reactions were noted to result from PGA degradation products for 10 weeks following fixation of malleolar fractures [368]. PGA implants have also been shown to induce the activation of the compliment system indicating a localized tissue reaction due to the acidic nature of degradation products [370]. In general, PLA-PGA copolymers demonstrate satisfactory biocompatibility with bone, and absence of significant toxicity, although some reduction in cell proliferation and inflammatory responses has been reported [371]. Biocompatibility and absence of infection or inflammation have been observed in studies to promote articular healing in osteochondral defects in the rabbit [372]. Early studies conducted on PLLA implanted in dog femurs have indicated that particles released from these polymers can impede bone formation after 6 weeks by inducing foreign-body inflammatory reactions [373]. However, PLLA-PGA implanted in rabbit skulls has been seen to degrade after 1 year without long-term implications even if inflammation was evident up to 9 months after implantation [374]. After 1 year of implantation, the broken down PLLA is replaced by a comparatively avascular granular fibrous tissue and, after 3 years of implantation, this tissue remains [375]. No inflammatory or foreign body reaction was observed in response to implantation of ultra-high strength l-PLA rods for up to 12 months in the medullary cavity of rabbit femora [376]. When l-PLA was used for a meniscal reconstruction in a dog study, presence of macrophages, fibroblasts, giant cells and lymphocytes were observed [377]. It seems that biocompatibility is compromised once degradation is in full swing and the small particles released promote a foreign body inflammatory reaction, as described in a study where l-PLA was implanted in femoral bones in dogs [378]. Macrophage-like cells and small LPLA particles have also been found in lymph nodes, in a study examining implant materials in the goat femoral diaphysis [379].

The inflammatory response to polymer degradation can be controlled somewhat by the incorporation of basic salts such as sodium bicarbonate, calcium bicarbonate and calcium hydroxyapatite [380]. Also, the incorporation of TCP [381], HA [382] and basic salts [228] into the polymeric matrix results in the production of a hybrid/composite material. These inorganic filler inclusions tailor the degradation and resorption kinetics of the polymer matrix. Such composite materials demonstrate improved biocompatibility and hard tissue integration [383]. In addition, the basic resorption products of HA or TCP buffer the acidic resorption by-products of the aliphatic polyesters and prevent the pH from becoming too low [228,381,382]. More recently, nano-HA incorporated to PLGA scaffolds have been shown to reduce the inflammatory response [384]. Nevertheless, it has been suggested that slow degrading polymers such as PCL induce higher magnitude of angiogenesis when compared to more acidic, faster degrading materials such as PLGA [42]. Conversely, chitosan induces an acute inflammatory response characterized by migration of neutrophils to the implant site which resolves a 12 weeks after implantation. Furthermore, chitosan also induces angiogenesis with minimal chronic inflammation [383]. Gorzelanny *et al.* have shown that chitosan demonstrates very little inflammatory response upon enzymatic degradation [385].

Calcium phosphate based bioceramics are also widely used for bone regeneration applications. Biodegradable dicalcium phosphates (brushite and monetite) are generally well tolerated by bone and soft tissues and do not cause inflammations in the long-term [386,387]. Following implantation these cements are enclosed in loose connective tissue [388], although they can also be surrounded by fibrous connective tissue if the cement composition is acidic [128]. *In vivo* studies have shown that early resorption of calcium phosphate cements is regulated by macrophages rather than osteoclasts [389,390]. Similar to *in vitro* studies implanted cement grafts can resorb via disintegration/fragmentation and rather passive dissolution based upon the solubility constant product of the material [391]. This is critical, since it is known that particles released from calcium phosphate cements can affect osteoblast function, viability, proliferation and production of extracellular matrix adversely [44]. The maximum number of particles that a single osteoblast can support is ~50, and the smaller the disintegration products are, the stronger the negative effect is observed [392].These released particles can also potentially result in peri-implant osteolysis and failure if the micro-environment around the implanted biomaterial is not cleared by extra-cellular media refreshment [393].

## 4. Biodegradation of Implanted Materials and Bone Tissue Formation

The importance of biomaterial degradation (both the rate and extent) cannot be overstated for bone repair and regeneration applications. The degradation capability of biomaterials implanted allows for space to be produced for newly forming bone tissue to not only grow along the implant surface (creeping substitution via osteoconduction) but also to infiltrate within the resorbing cement matrix along with new blood vessels [38]. This infiltration of biomaterial scaffold matrix with blood vessels allows for the bone formation front to progress and be provided with oxygen that is mandatory for survival of the regenerating tissues [394]. It has been observed that some fractured bone tissues can heal within a period of 10–18 months although this varies with the type of bone and function [371]. It is crucial for the biodegradable scaffold to retain its strength during the healing period so as to provide fixation at the fracture site but degrade after the healing as completed. Generally, polymers of the poly(α-hydroxy acids) group undergo bulk degradation. Upon placement in aqueous media it has been shown that the molecular weight of the polymer commences to decrease on day one for PGA and PDLA, or after a few weeks for PLLA. However, the mass loss does not start until the molecular chains are reduced to a size which allows them to freely diffuse out of the polymer matrix and similar process occurs after implantation [395]. As seen in Table 1, d,l(PLA) and l(PLA), two biodegradable polymers employed for fracture fixation, degrade after 12–16 months and 24 months respectively. This makes polymers a promising material choice for fracture fixation (provided they have adequate mechanical properties) as far as degradation and healing times are concerned; by the time the fracture heals, the polymers would have degraded completely.

Initial resorption of calcium phosphate cement grafts is affected by the inherent cement properties such as porosity, as well as the site of implantation, which affects the rate of fluid exchange and the properties of the surrounding medium [318,391,396]. The amount of new bone formed is also highly dependent on implantation site and vascular supply, as an adequate blood supply increases the speed of cement resorption and replacement by new woven bone [389]. It is known for serum proteins to be adsorbed onto the cement surface, altering the interfacial properties of the calcium phosphate crystals [397], and favoring *in vivo* resorption [391]. Research shows that unlike HA cements that undergo negligible resorption over time, dicalcium phosphate cements resorb to a much greater extent *in vivo* [386,398]. Following implantation, they appear to be rapidly resorbed by simple dissolution and cellular activity [318,399], although the later seems to be the more predominant factor [400]. These cements exhibit an increase in porosity, a decrease in mass and deterioration in mechanical properties [401]. It has been shown that brushite cements experience an initial linear degradation rate of 0.25 mm per week [402]. This overwhelms the bone formation capacity, resulting in a small bone-material gap and a reduction in the graft mechanical properties [403]. However, after a few weeks implantation the mechanical properties improve, due to bone in-growth into the biomaterial scaffold matrix [44,403]. After the fast degradation of the implanted cements initially, the remaining cement matrix is converted into less soluble apatite via phase transformation and re-precipitation [44]. This results in the resorption of the remaining cement to become very slow and limits the extent of degradation and ultimately bone formation and in-growth [404]. After 24 weeks of implantation in an animal model (sheep), brushite cements have been shown to completely convert to poorly crystalline carbonated apatite [405]. At this point there is almost no passive dissolution of the cement that occurs, and resorption is dependent entirely upon osteoclastic activity, rather than macrophage mediated phagocytosis [389,400]. The composition of brushite is seen to be stable when stored in distilled water and shows no conversion to apatite [406] also, when stored under alkaline conditions, brushite cements are not converted into apatite unless organic biomolecules (e.g., 10 mM citrate) are added. This indicates that the interfacial energy barrier between the brushite–solution and apatite-solution interfaces in too high to allow spontaneous conversion. However, with the addition of citrate ions, a significant reduction in this energy barrier is observed, and results conversion to HA *in vivo* [407]. Similar effects have been observed with other polymeric additives such as hyaluronic acid and collagen that slightly decreases the cement resorption rate *in vivo* [408].

As mentioned earlier, the resorption of cement matrix is an important feature with respect to bone formation at the implanted sites, since it frees up the space needed for new bone formation ideally without compromising mechanical stability. This is the reason that the amount of bone regenerated when using dicalcium phosphate materials is usually higher than that obtained with non-resorbable biomaterials such as HA [125,386,409]. The surfaces of bioceramics such as brushite and monetite have been shown to stimulate osteoblasts activity [410]. Cell culture studies performed on magnesium-doped brushite cements have revealed increased cell proliferation and differentiation [411]. Also, certain polymeric additives, such as collagen, improve cell adhesion to brushite [123], while xanthan gum has a negative effect on the biological response of the cement, resulting in less bone being formed and greater formation of fibrous tissue [408]. The release of growth factors incorporated into cement matrices has also been used to stimulate the bone formation. Vascular endothelial growth factor (VEGF), platelet-derived growth factor (PDGF) and receptor activator of nuclear factor jB ligand (RANKL) are some of the growth factors that have been assessed to enhance bone regenerative capacity *in vivo* [44,412]. Bone formation has been observed to be considerably greater with PDGF-loaded brushite–chitosan scaffolds, as well as with the combination PDGF/VEGF [413]. RANKL is a growth factor that promotes osteoclast differentiation and is important towards biodegradation calcium phosphate grafts [414]. Results from studies suggest that the application of growth factors using biodegradable materials could improve the tissue response and promote bone formation in bone regeneration applications [412].

## 5. Importance of Physical Properties and Geometrical Considerations of Biodegradable Scaffolds Used for Bone Tissue Engineering

Various fabrication techniques are applied to process biodegradable materials into 3D polymeric and bioceramics scaffolds with differing geometry affecting physical properties (e.g., porosity and surface area) [67,220,298,415,416]. It is imperative that the created 3D scaffold have and maintain sufficient structural integrity during the bone regeneration and remodeling process [417]. Bioceramics are weak under tension and stronger under compression and these facts need to be taken into consideration when fabricating pre-set block grafts for bone tissue engineering applications [418]. Polymers on the other hand provide an opportunity to be prepared into scaffolds with varying geometries, thickness and internal configurations. The physical scaffold structure is required to support the polymer/cell/bone tissue construct from the time of implantation up to the point where remodelling occurs by the host tissue. In the case of load-bearing situations, the scaffold matrix is required to serve an additional function by providing sufficient temporary mechanical support to withstand *in vivo* stresses and physiological loading [43]. Therefore, the biomaterial must be selected and then the scaffold designed with an *in vivo* degradation rate such that the strength of the scaffold is retained until the tissue engineered transplant is fully remodeled and ultimately assumes its structural role. Also, it is desirable for the mechanical properties of the created scaffold to match that of the host tissue as closely as possible at the time of implantation [380].

It has been noted that under cyclic compressive loading, the polymer matrix of PLGA initially collapses and then stiffens as suggested by the changes in surface deformation and morphology [419]. Another way of designing 3D scaffold constructs are by applying the concept of tensegrity, which evenly distributes and balances mechanical stresses [420,421]. This is achieved by connecting the scaffold framework made up of walls and struts into triangles, pentagons or hexagons, each of which can bear tension or compression. Aligned electrospun collagen fibers have shown to decrease cellular adhesion but a higher cellular proliferation when compared to random fibers [422]. Furthermore, changing the fiber orientation also helps to control the direction of cellular proliferation which can be significantly advantageous when these fibers are used as scaffolds [423]. While it difficult to control the fiber diameter and porosity of electrospun scaffolds at the microscopic level [424], rapid prototyping makes it possible to produce scaffolds with a specific pore and fiber geometry at micro- as well as a macroscopic level [425]. It has been observed that scaffolds produced by rapid prototyping, possessing an average pore size that progressively decreases in the outer layers, have intermediate elastic properties when compared to those possessing a uniform pore-size [426]. It has been seen that decreasing the fiber width and the thickness of layers increases the stiffness of scaffolds [427]. Moreover, producing scaffolds with a higher porosity can decrease the Young modulus [428]. The aforementioned research suggests that scaffolds produced by rapid prototyping can be tailor-made to suit specific implantations sites such as cartilage, tendon and bone which have very different mechanical and physical properties when compared with each other [425].

In order to tissue engineer bone, the creation of a vascularized bed ensures the survival and function of the 3D scaffold/tissue construct by providing nutrition, gas exchange, and elimination of by-products [429]. Since the distance between blood vessels and mesenchymal cells are not larger than 100 µm *in vivo* [430], vascularization of a scaffold may not be achieved by purely relying on capillary ingrowth into the interconnecting pore network from the host tissue. Hence, a porous network structure is necessary in a scaffold to optimize cellular proliferation and nutrient flow. Having an interconnected macropore-structure of 300–500 µm enhances the diffusion rates to and from the center of a scaffold, however, the passage of nutrients and by-products might not occur sufficiently nor efficiently when larger scaffold volumes are employed [298]. The use of pre-vascularization [431], and/or arterio-venous (AV) loops [432], can result in creation of a fluid dynamic microenvironment within the implanted macroporous scaffold that mimics the interstitial fluid conditions present in natural bone [433]. It is also possible to accelerate the rate of vascularization by incorporating angiogenic factors in the degrading matrix of the scaffold [434]. The time frame also has to be taken into account for the capillary system to distribute through larger scaffold volume before degradation start and disintegration of the graft material occurs.

The presence of macroporosity in bioceramic scaffolds used for bone repair and regeneration is important in allowing cellular infiltration and proliferation inside the biomaterial [435]. However, increasing the porosity can potentially affects the mechanical properties adversely as mechanical strength of cements is inversely proportional to their porosity. Therefore, the incorporation of macro-pores within the cement structure has to be performed without increasing the overall cement porosity. This can be done by adding porogens, such as mannitol, which create pores having width of 250–500 µm in bioceramics scaffolds without reducing the initial compressive strength of the cement [436]. Another way of creating macroporosity in is by using gelatin powder as a template, which produces a closely packed structure with open pores of 100–200 µm [382]. However, the limiting factor of using these techniques is the lack of interconnectivity of the pores created. Better control over pore geometry and distribution can be achieved via computer aided design (CAD) of 3D printed brushite bioceramics [44,138]. CAD allows for specific pore designs to be included with varying geometries of the pores incorporated [437].

## 6. Conclusions

The development of biomaterials for bone repair devices and prostheses is a challenge from an engineering and biological perspective. In the field of biomaterials research, degradable materials for bone repair and regeneration are actively sought and generate a lot of interest since their biodegradable nature allows avoiding the second surgery and reduction in the pain and cost for patients. Natural and synthetic polymers and bioceramics are already in clinical use as biodegradable materials and magnesium based metals are a new class of biodegradable materials in development. The mechanical properties, biological behavior and biodegradation mechanism vary for different biomaterials. In comparison with polymers and bioceramics, the tensile strength and stress elongation of magnesium alloys is higher. The highest level brittleness is exhibited by the ceramic materials. From a biological perspective, it has been shown that more new bone is formed around bioceramics and magnesium alloys than around polymers. This can be attributed to the osteoconductive and at times osteoinductive properties the ceramics possess and also the bioactive behavior of magnesium alloys. The acidic degradation products of various polymeric materials can frequently induce inflammatory response which is not observed with the use of bioceramics. Degradation rate and extent is one of the most important characteristics for degradable biomaterials. Bioceramics degrade and show *in vivo* resorption by cell-mediated and solution-driven processes and demonstrate progressive replacement by lamellar true bone. Biodegradable polymers mostly degrade by enzymolysis and hydrolysis from macromolecules to smaller molecules, and eventually to carbon dioxide and water. The mechanical strength decreases slowly at the initial stage of polymeric degradation, and rapidly during bulk degradation. Metals and alloys that are based on magnesium as their component degrade by corrosion in body fluid with comparatively high degradation rate at the initial stage that becomes progressively slower with time. The mechanical strength of magnesium alloys does not decrease during degradation since their inner structures remain unchanged. Conventional metallic prosthesis constructed using non-degradable materials are fast becoming obsolete due to their inherent disadvantages. As the review indicates, the three major kind of biodegradable materials have various advantages and limitations which need to be recognized prior to being selected for the applications they are intended for. Biodegradable and bioactive composite materials are being researched for the creation of high performance implant materials for osteologic repair applications. It is expected that the next generation of biodegradable materials will demonstrate vast improvements in implant and biological tissue interfacing based on the knowledge gained from recent research. However, extensive work is required in order to obtain the ideal bone repair and regeneration biomaterials in the future.
